# Sex differences in clinical characteristics and long-term outcomes in patients with vasospastic angina: results from the VA-Korea registry, a prospective multi-center cohort

**DOI:** 10.1186/s13293-020-00341-4

**Published:** 2020-11-23

**Authors:** Hack-Lyoung Kim, Sang-Ho Jo, Hyun-Jin Kim, Min-Ho Lee, Won-Woo Seo, Sang Hong Baek

**Affiliations:** 1 Division of Cardiology, Department of Internal Medicine, Boramae Medical Center, Seoul National University College of Medicine, Seoul, Korea; 2 Division of Cardiology, Department of Internal Medicine, Hallym University Sacred Heart Hospital, Anyang, Korea; 3 Department of Cardiology in Internal Medicine, Hanyang University Guri Hospital, Guri, Korea; 4 Department of Cardiovascular Medicine, Soonchunhyang Seoul Hospital, Seoul, Korea; 5 Department of Cardiovascular Medicine, Hallym University Kangdong Hospital, Seoul, Korea; 6 Department of Cardiovascular Medicine, Seoul St. Mary’s Hospital, The Catholic University of Korea, Seoul, Korea

**Keywords:** Clinical characteristics, Prognosis, Sex difference, Vasospastic angina

## Abstract

**Background:**

Sex differences in clinical characteristics and prognosis of vasospastic angina (VA) have not been well elucidated. This study was performed to investigate sex-specific characteristics and predictors for long-term clinical outcomes in patients with VA.

**Methods:**

We analyzed 1838 patients (55 years and 62% male) who were diagnosed with definite (*n* = 680) or intermediate (*n* = 1212) VA in ergonovine provocation test from a nation-wide VA registry. The primary study end-point was composite events including cardiac death, acute coronary syndrome, ventricular tachycardia or fibrillation, and atrioventricular block during clinical follow-up.

**Results:**

Male patients were younger, and there were more smokers and alcohol drinkers in male patients than in female patients. During the median follow-up period of 760 days (interquartile range, 336–1105 days), there were 73 cases (3.97%) of composite events. There was no sex difference in the occurrence of composite events (log-rank *p* = 0.649). Concomitant significant (≥ 50%) organic coronary stenosis was associated with worse clinical outcomes in both male (hazard ration [HR], 1.97; 95% confidence interval [CI], 1.01–3.85; *p* = 0.047) and female (HR, 3.26; 95% CI, 1.07–9.89; *p* = 0.037) patients. Obesity (body mass index ≥ 25 kg/m^2^) was associated with better prognosis in female VA patients (HR, 0.22; 95% CI, 0.07–0.68; *p* = 0.008). Even when only patients with definite diagnosis of VA were considered, there was no significant sex difference in clinical outcomes (log-rank *p* = 0.876).

**Conclusions:**

In VA patients, there were several different clinical characteristics according to sex; however, long-term clinical outcome was similar between sexes. Significant organic coronary stenosis in both sexes and low body mass index (< 25 kg/m^2^) in females were associated with worse prognosis in VA patients.

## Introduction

Vasospastic angina (VA) is characterized by recurrent chest pain, not related to exertion, with dynamic ST segment changes in electrocardiograms, which is caused by coronary artery spasm [1–3]. In general, VA is known to have a better long-term prognosis compared to atherosclerotic coronary stenosis due to its good response to vasodilators [4–6]. In some cases, however, VA can cause fatal complications such as acute myocardial infarction and ventricular arrhythmia leading to sudden cardiac death; thus, vasodilator therapy should not be neglected [7, 8].

Over the past decade, effort to understand sex differences has continued, and realized the importance of cardiovascular disease in females [9, 10]. In particular, sex differences in coronary vasomotor function are relatively well known. It has been suggested that increased coronary reactivity and microvascular dysfunction play significant roles in ischemic heart disease in females [10–12]. VA is also caused by the functional abnormalities of the coronary artery; however, sex differences in VA have not yet been well studied. In particular, VA is highly prevalent in males [1, 2], and there has been limited data on the characteristics of female patients with VA. Until now, only a few studies have addressed sex difference in VA [13–17]. Among these studies, only two studies have investigated sex differences in long-term VA prognosis; however, sex-specific factors determining long-term clinical outcome of VA are inconsistent [13, 15]. Therefore, using a large number of VA patients from the prospective nation-wide registry database, this study was performed to elucidate more clearly sex differences in clinical characteristics, long-term clinical outcomes of VA, and to find out sex-related risk factors associated with VA prognosis.

## Methods

### Study patients

Data was from the nation-wide prospective registry of VA in Korea (VA-Korea) [18, 19]. Eleven tertiary hospitals in South Korea participated in this registry. Between May 2010 and June 2015, consecutive patients (> 18 years) with suspected VA and underwent invasive coronary angiography (CAG) and ergonovine (EG) provocation test were enrolled. Patients who had a normal or mild (< 50% luminal diameter narrowing) coronary atherosclerotic stenosis at baseline CAG were eligible. However, EG provocation test was also performed in some patients with intermediate (50–70%) organic stenosis by the attending physician’s discretion when VA was strongly suspected. Patients having malignancy, end-stage renal disease on dialysis, and inflammatory disease were excluded [20]. Initially 2960 patients with suspected VA were enrolled, and 868 patients with negative results in EG provocation test were excluded. After further exclusion of 254 patients with missing data and loss of clinical follow-up, the remaining 1838 patients were analyzed in this study. The study protocol complied with the Declaration of Helsinki, and was approved by the Institutional Review Board of each participating hospital. Written informed consent was obtained from each patient.

### Data collections

Data was collected from the VA-Korea registry database through a web-based electronic data-capture system containing an electronic case report form. Body mass index (BMI) was calculated by dividing body weight in kilograms by the square of height in meters (kg/m^2^). Information on cardiovascular risk factors was obtained, which included hypertension, diabetes mellitus, dyslipidemia, cigarette smoking, and alcohol drinking. The blood levels of important biochemical variables, including hemoglobin, creatinine, glucose, total cholesterol, low-density lipoprotein cholesterol, high-density lipoprotein cholesterol, and triglyceride at admission, were identified. Left ventricular ejection fraction was obtained using Simpson’s biplane method during transthoracic echocardiography. Cardiovascular medications, including antiplatelets, calcium channel blockers, renin-angiotensin system blockers, beta-blockers, vasodilators, and statin were also assessed.

### Invasive CAG and EG provocation test

**Fig. 1:**
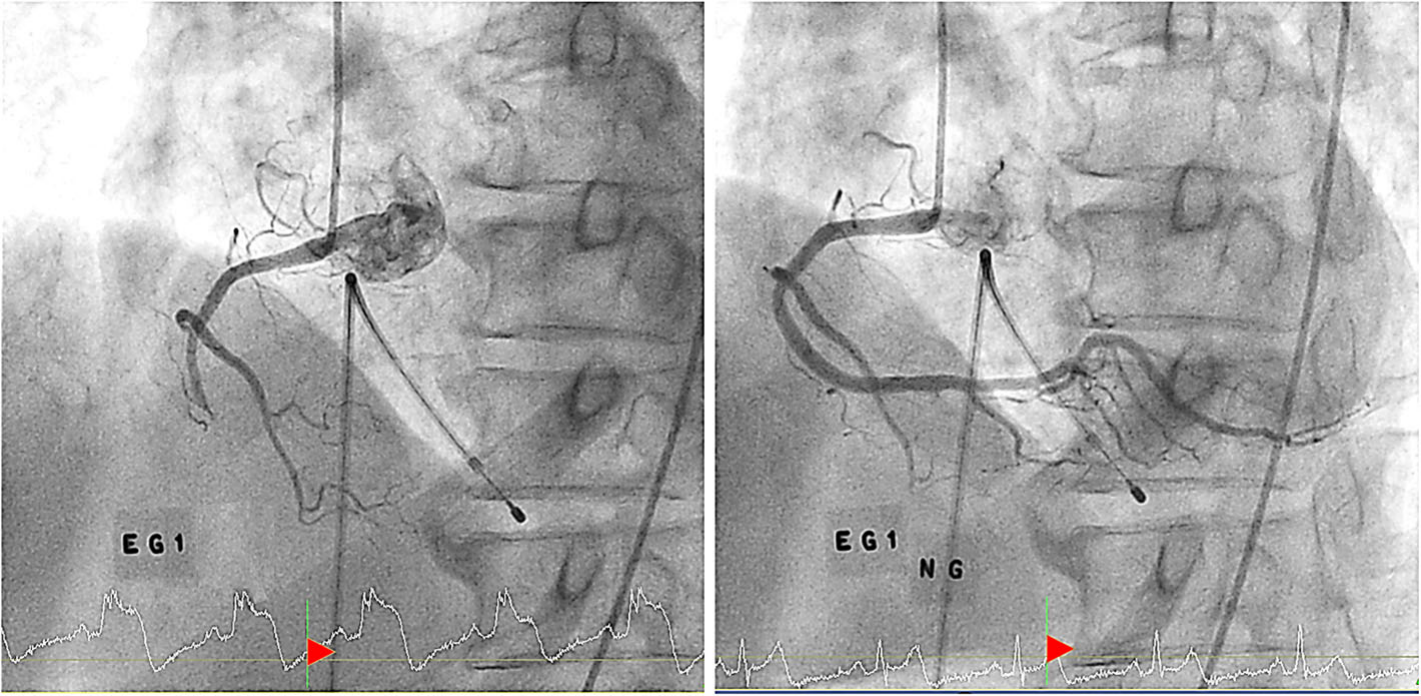
Coronary angiographic findings of a typical case of vasospastic angina. (Left figure) Angiography of right coronary artery shows totally occluded coronary artery after the first dose of intracoronary ergonovine (EG1). At the bottom of the image, ST segment elevation was notable at the time of coronary artery spasm. (Right figure) After injection of nitroglycerin into coronary artery (NG), coronary spasm was relieved and normal coronary artery was visualized. ST segment elevation was also normalized after nitroglycerin injection

CAG was performed by well-trained cardiologists according to the standardized method [21]. Vasoactive medications were stopped at least 48 h before CAG. CAG was performed via the right femoral artery without the use of nitroglycerin. In cases of no significant atherosclerotic coronary stenosis on CAG, EG provocation test was performed using the same protocol in all participating hospitals [18, 20, 22]. In some patients, even with significant narrowing of the coronary arteries, EG provocation was performed when the lesions did not match the clinical feature or they were suspected of accompanying VA. Provocation test was started with the right coronary artery (RCA) and then the left coronary artery (LCA). EG was diluted in saline and injected slowly into the coronary arteries over 2–3 min. If coronary spasm was not induced, EG doses were gradually increased from 10 to 20 μg and 40 μg during the RCA angiography, and from 20 to 40 μg and 60 μg during the LCA angiography. Once coronary spasm was provoked, 200 μg of nitroglycerin was immediately injected into coronary artery. Chest pain and electrocardiographic (ECG) changes were also recorded during the procedure. Definite VA was defined as a total (100%) or subtotal (> 90% luminal diameter narrowing) occlusion of the index coronary artery accompanied by ischemic symptoms and/or ECG changes. An ischemic ECG change was defined as an ST segment elevation or depression > 0.1 mV or a negative U-wave in at least two contiguous leads [22]. An intermediate result was defined as 50–90% luminal narrowing with or without ischemic symptoms and/or ECG changes. A negative result was defined as both RCA and LCA EG provocation tests with < 50% luminal narrowing without ischemic symptoms nor ECG changes. Cather-induced spasm was excluded. The CAG results were analyzed in detail by dividing each coronary artery and its segments. Angiographic findings were analyzed on-line or off-line using a dedicated quantitative coronary angiography program or manual assessment by investigators in each hospital who were not involved in the study. In addition, investigators at the core laboratory of Seoul St. Mary’s Hospital in South Korea confirmed the angiographic data off-line by visual assessment in a blinded fashion [18]. Significant organic stenosis of the coronary artery was defined as luminal diameter narrowing ≥ 50% that did not respond to intracoronary nitrate infusion. Angiographic finding of typical case of VA is demonstrated in Fig. 1.

### Clinical events

Cardiovascular events were assessed during the follow-up. The primary endpoint was a composite of cardiac death, acute coronary syndrome, ventricular tachycardia or fibrillation, and atrioventricular block. Cardiac death was defined as sudden unexplained death, and death caused by acute coronary syndrome, pump failure, and cardiac arrhythmia [18, 19]. Acute coronary syndrome included acute myocardial infarction and unstable angina, which were diagnosed based on typical ischemic symptoms and ECG changes with (acute myocardial infarction) or without (unstable angina) cardiac enzyme elevation [23]. Ventricular tachycardia or fibrillation and atrioventricular block were confirmed in ECG.

### Statistical analysis

Continuous variables are expressed as mean ± standard deviation, and categorical variables as percentages. Continuous variables were compared using Student’s *t* test, and categorical variables using the Chi-square test between sexes. Event-free survival rates between the two groups were demonstrated using Kaplan-Meier survival curves and compared using the log-rank test. Independent risk factors for composite events were analyzed using Cox proportional hazard regression analysis. The following variables were considered to be potential confounders, and were controlled during the multivariable analyses: age, BMI, hypertension, diabetes mellitus, cigarette smoking, significant atherosclerosis, spasm severity, and the use of calcium channel blockers. A *p* value of < 0.05 was considered statistically significant. All statistical analyses were conducted using the Statistical Package for the Social Science (SPSS) 20.0 (IBM Co., Armonk, NY, USA).

## Results

### Baseline clinical characteristics

The mean age of total study patients (*n* = 1838) were 55.1 ± 11.3 years, and 1141 (62.1%) were male. The baseline clinical, ECG, and angiographic characteristics of the study patients according to sex are shown in Table 1. Male patients were younger and had higher systolic and diastolic blood pressures compared to female patients. Cigarette smoking and alcohol drinking were more frequently observed in male patients than in female patients. Although statistical significance was not reached, more cardiac arrest (*p* = 0.091) and syncope (*p* = 0.055) appeared in male. Compared to female patients, male patients had higher levels of hemoglobin, creatinine, glucose, and triglyceride, and had lower levels of total cholesterol, LDL cholesterol, and HDL cholesterol in blood. Left ventricular ejection fraction was within the normal limit and similar in both sexes. In ECG findings, ST segment elevation was more frequently observed in male patients, and T inversion was in female patients. In CAG findings, significant organic stenosis of the coronary artery was more frequently detected in male patients compared to female patients. The rate of definite diagnosis of VA by using EG provocation test was significantly higher in male patients than in female patients (43.2% vs. 25.5%, *p* < 0.001). Among discharge medications, antiplatelets, RAS blockers, nicorandil, and statin were more frequently prescribed in male patients, and trimetazidime in female patients.

**Table 1:** Characteristics of study patients

Characteristic	Male (*n* = 1141)	Female (*n* = 697)	*p* value
Age, years	54.0 ± 11.5	56.9 ± 10.6	< 0.001
Body mass index, kg/m^2^	24.7 ± 3.02	24.7 ± 3.8	0.839
Systolic blood pressure, mmHg	127 ± 18	123 ± 19	< 0.001
Diastolic blood pressure, mmHg	78.2 ± 12.4	75.0 ± 11.7	< 0.001
Cardiovascular risk factor			
Hypertension	437 (38.3)	260 (37.3)	0.659
Diabetes mellitus	121 (10.6)	56 (8.0)	0.067
Dyslipidemia	176 (15.5)	124 (17.8)	0.188
Previous CAD	147 (12.9)	74 (10.6)	0.142
Current smoking	465 (41.5)	37 (5.4)	< 0.001
Alcohol drinking	655 (57.4)	107 (15.4)	< 0.001
Clinical diagnosis			
Angina	1,036 (90.9)	639 (91.9)	0.433
Acute myocardial infarction	25 (2.2)	9 (1.3)	0.166
Cardiac arrest	21 (1.8)	6 (0.9)	0.091
Syncope	18 (1.6)	4 (0.6)	0.055
Ventricular tachycardia or fibrillation	9 (0.8)	3 (0.4)	0.552
Atrioventricular block	1 (0.1)	0	0.999
Laboratory finding			
Hemoglobin, g/dL	14.5 ± 1.5	12.8 ± 1.2	< 0.001
Creatinine, mg/dL	0.88 ± 0.38	0.64 ± 0.16	< 0.001
Glucose, mg/dL	114 ± 39	106 ± 35	< 0.001
Total cholesterol, mg/dL	171 ± 36	178 ± 36	< 0.001
LDL cholesterol, mg/dL	101 ± 31	106 ± 31	0.004
HDL cholesterol, mg/dL	44.3 ± 12.1	50.3 ± 13.0	< 0.001
Triglyceride, mg/dL	156 ± 111	122 ± 89	< 0.001
Left ventricular EF, %	64.2 ± 6.8	64.8 ± 6.3	0.073
ECG finding			
Normal sinus rhythm	1,013 (88.9)	646 (92.7)	0.007
Atrial fibrillation or atrial flutter	19 (1.7)	10 (1.4)	0.699
Ventricular tachycardia	2 (0.2)	0	0.529
Ventricular fibrillation	8 (0.7)	3 (0.4)	0.549
Atrioventricular block	13 (1.1)	7 (1.0)	0.999
ST segment elevation	93 (8.2)	9 (1.3)	< 0.001
ST segment depression	12 (1.1)	5 (0.7)	0.468
T inversion	21 (1.8)	25 (3.6)	0.020
CAG finding			
Atherosclerosis of total coronary artery			
Any	448 (39.3)	191 (27.5)	< 0.001
Significant (> 50%)	159 (13.9)	60 (8.6)	0.001
LM atherosclerosis			
Any	14 (1.2)	5 (0.7)	0.296
Significant (> 50%)	0	0	1.000
Mean stenosis	28.5 ± 6.3	24.0 ± 5.4	1.670
LAD atherosclerosis			
Any	304 (26.7)	140 (20.1)	0.002
Significant (> 50%)	100 (8.8)	41 (5.9)	0.025
Mean stenosis	36.7 ± 16.9	36.2 ± 16.0	0.801
LCX atherosclerosis			
Any	142 (12.5)	47 (6.8)	< 0.001
Significant (> 50%)	38 (3.3)	10 (1.4)	0.014
Mean stenosis	37.1 ± 16.9	33.0 ± 16.0	0.203
RCA atherosclerosis			
Any	219 (19.2)	89 (12.8)	< 0.001
Significant (> 50%)	54 (4.7)	16 (2.3)	0.008
Mean stenosis	36.4 ± 18.3	32.2 ± 14.9	0.039
Result of ergonovine provocation test			< 0.001
Definite	493 (43.2)	178 (25.5)	
Intermediate	648 (56.8)	519 (74.5)	
Discharge medication			
Antiplatelet	574 (50.3)	287 (41.2)	< 0.001
Calcium channel blocker			
Dihydropyridine	258 (22.6)	133 (19.1)	0.073
Non-dihydropyridine	864 (75.7)	531 (76.2)	0.823
RAS blocker	228 (20.0)	101 (14.5)	0.003
Beta-blocker	68 (6.0)	49 (7.0)	0.362
Nitrate	216 (18.9)	128 (18.4)	0.763
Nicorandil	442 (38.7)	235 (33.7)	0.030
Molsidomine	167 (14.6)	102 (14.6)	0.999
Trimetazidime	103 (9.0)	101 (14.5)	< 0.001
Statin	576 (50.5)	315 (45.2)	0.028

Numbers are represented as mean ± SD or *n* (%). *CAD* Coronary artery disease, *LDL* Low-density lipoprotein, *HDL* High-density lipoprotein, *EF* Ejection fraction, *ECG* Electrocardiogram, *CAG* Coronary angiography, *LM* Left main, *LAD* Left anterior descending artery, *LCX* Left circumflex artery, *RCA* Right coronary artery, *RAS* Renin-angiotensin system

### Clinical events

**Fig. 2:**
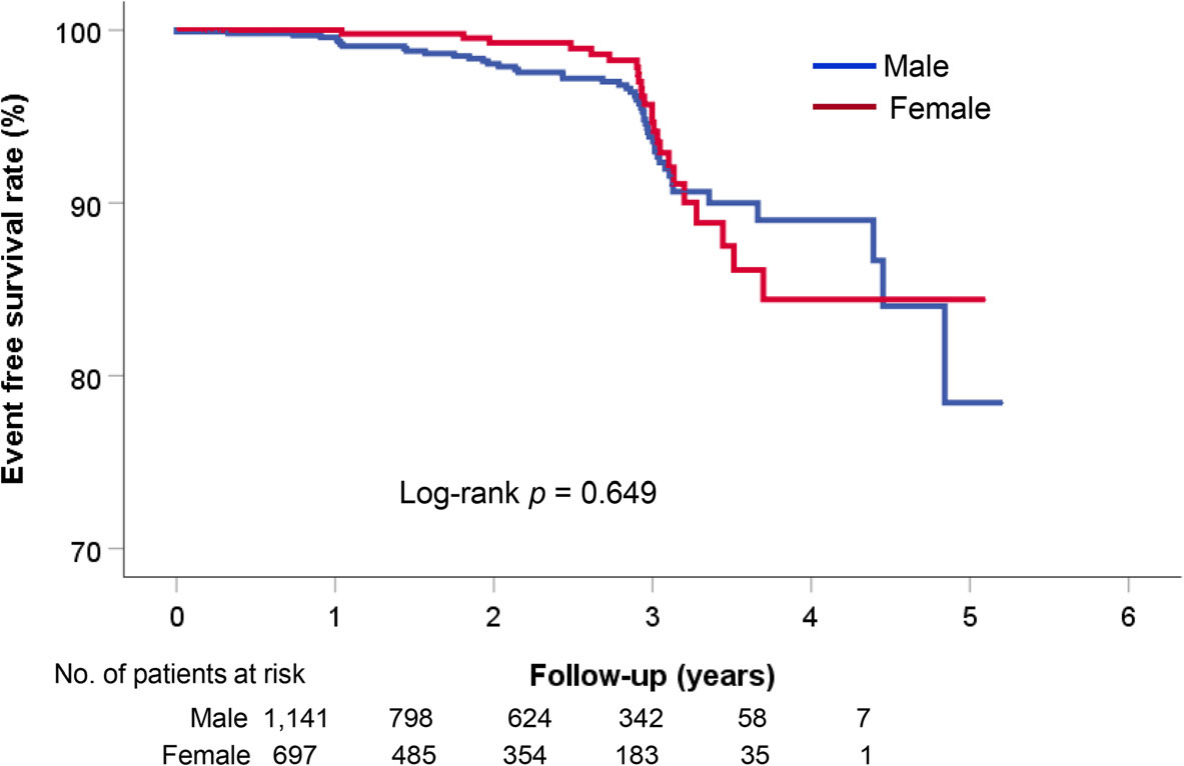
Sex-specific long-term clinical outcomes in patients with vasospastic angina

**Fig. 3:**
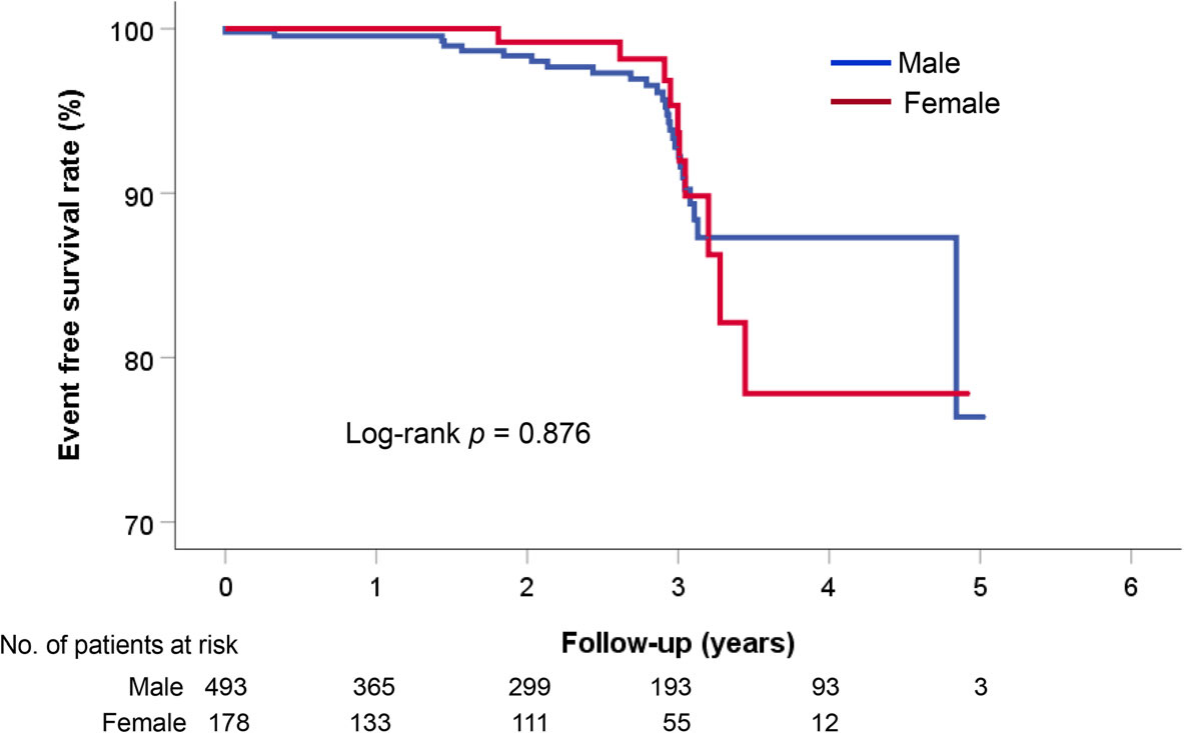
Sex-specific long-term clinical outcomes in patients with definite diagnosis of vasospastic angina

**Fig. 4:**
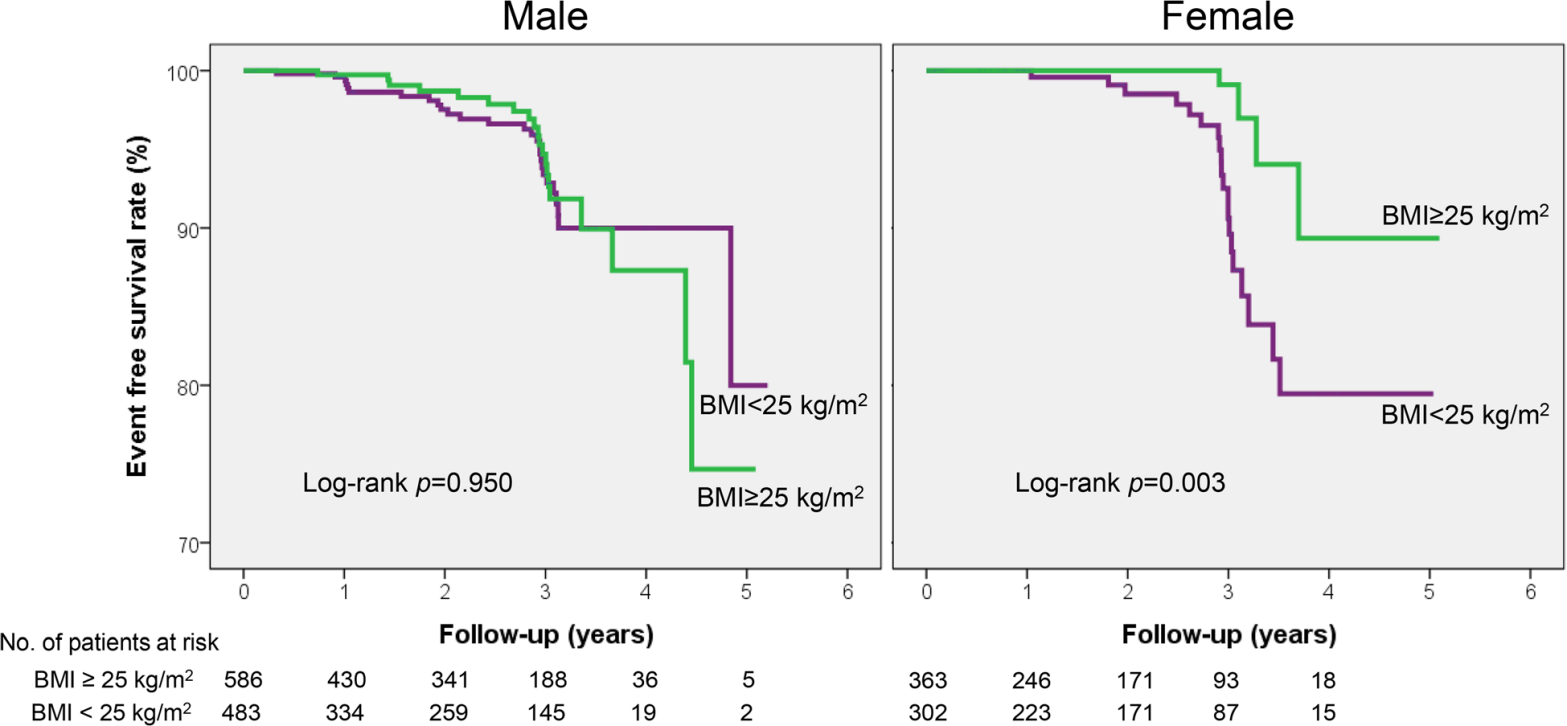
Long-term clinical outcomes according to BMI in male and female patients with vasospastic angina. *BMI* body mass index

The occurrence of clinical events is demonstrated in Table 2. During the median follow-up period of 760 days (interquartile range, 336–1105 days), there were 13 all-cause death (7.01%), 5 cardiac death (0.27%), 57 acute coronary syndrome (3.10%), 7 ventricular tachycardia/fibrillation (0.38%), and 6 atrioventricular block (0.32%). Composite events occurred in 73 cases (3.97%). In sex comparisons, cardiac death (*p =* 0.164) and ventricular tachycardia/fibrillation (*p* = 0.049) occurred in only male patients; the incidences of other events were similar between sexes (*p* > 0.05 for each). Independent risk factors for composite events are shown in Table 3. The presence of significant organic stenosis was independently associated with both male (hazard ration [HR], 1.97; 95% confidence interval [CI], 1.01–3.85; *p* = 0.047) and female (HR, 3.26; 95% CI, 1.07–9.89; *p* = 0.037) patients. The Kaplan-Meier event-free survival curve according to sex is demonstrated in Fig. 2. Even when only patients with definite diagnosis of VA were considered, there was no significant sex difference in clinical outcomes (log-rank *p* = 0.876) (Fig. 3). In female patients, higher BMI (≥ 25 kg/m^2^) was associated with lower risk of composite events (HR, 0.22; 95% CI, 0.07–0.68; *p* = 0.008). Other factors such as age, cardiovascular risk factors, cigarette smoking, and the use of calcium channel blockers were not associated with VA outcomes in both sexes (*p* > 0.05 for each). Kaplan-Meier event-free survival curves according to BMI and sex are demonstrated in Fig. 4. Characteristics of female patients with VA according to BMI are shown in Table 4.

**Table 2:** Clinical events

Clinical event	Male (*n* = 1141)	Female (*n* = 697)	*p* value
All-cause death	7 (0.6)	5 (0.7)	0.697
Composite events	49 (4.3)	24 (3.4)	0.365
Cardiac death	5 (0.4)	0	0.164
Acute coronary syndrome	35 (3.1)	22 (3.2)	0.915
Ventricular tachycardia or fibrillation	7 (0.6)	0	0.049
Atrioventricular block	3 (0.3)	3 (0.4)	0.679

Numbers are represented as *n* (%)

**Table 3:** Independent risk factors for cardiovascular events

Variable	Male		Female	
	HR (95% CI)	*p* value	HR (95% CI)	*p* value
Age ≥ 65 years	0.85 (0.38–1.89)	0.698	1.37 (0.52–3.58)	0.519
Body mass index ≥ 25 kg/m^2^	1.07 (0.58–1.95)	0.819	0.22 (0.07–0.68)	0.008
Hypertension	0.77 (0.41–1.42)	0.771	0.67 (0.25–1.76)	0.424
Diabetes mellitus	1.65 (0.73–3.72)	0.222	0.57 (0.07–4.47)	0.598
Current cigarette smoking	1.12 (0.62–2.02)	0.697	1.49 (0.36–6.68)	0.606
Significant atherosclerosis	1.97 (1.01–3.85)	0.047	3.26 (1.07–9.89)	0.037
Definite spasm	1.15 (0.64–2.08)	0.634	1.42 (0.60–3.31)	0.418
Non-dihydropyridine calcium channel blocker	2.25 (0.89–5.80)	0.091	2.09 (0.60–7.22)	0.242

*HR* Hazard ratio, *CI* Confidence interval

**Table 4:** Characteristics of female patients with VA according to BMI

Characteristic	BMI < 25 kg/m^2^ (*n* = 364)	BMI ≥ 25 kg/m^2^ (*n* = 303)	*p*
Age, years	56.3 ± 10.9	57.7 ± 10.4	0.036
BMI, kg/m^2^	22.2 ± 1.7	27.9 ± 3.4	< 0.001
Systolic blood pressure, mmHg	124 ± 19	123 ± 17	0.634
Diastolic blood pressure, mmHg	75.4 ± 12.3	74.1 ± 10.8	0.173
Cardiovascular risk factors			
Hypertension	111 (30.5)	141 (46.5)	< 0.001
Diabetes mellitus	16 (4.4)	37 (12.2)	< 0.001
Dyslipidemia	55 (15.2)	66 (21.9)	0.026
Current smoking	24 (6.6)	10 (3.3)	0.170
Alcohol drinking	66 (18.1)	36 (11.9)	0.026
Clinical diagnosis			
Acute myocardial infarction	5 (1.4)	4 (1.3)	0.960
Cardiac arrest	3 (0.8)	2 (0.7)	0.999
Syncope	2 (0.5)	2 (0.7)	0.999
Ventricular tachycardia or fibrillation	2 (0.5)	1 (0.3)	0.999
Significant atherosclerosis	29 (8.0)	27 (9.0)	0.642
Definite spasm	88 (24.2)	83 (27.4)	0.343
Laboratory findings			
Hemoglobin, g/dL	12.7 ± 1.2	12.9 ± 1.2	0.147
GFR, mL/min/1.73 m^2^	104 ± 48	100 ± 29	0.136
Glucose, mg/dL	105 ± 38	107 ± 31	0.502
Total cholesterol, mg/dL	178 ± 34	177 ± 37	0.710
LDL cholesterol, mg/dL	105 ± 30	107 ± 32	0.639
HDL cholesterol, mg/dL	52.4 ± 13.7	48.0 ± 11.7	< 0.001
Triglyceride, mg/dL	115 ± 101	132 ± 74	0.019
Left ventricular ejection fraction, %	64.4 ± 6.2	65.2 ± 6.4	0.126
Discharge medications			
Antiplatelet	138 (38.2)	141 (46.5)	0.030
Non-DHP calcium channel blocker	282 (77.5)	222 (73.3)	0.208
Nitrate	281 (77.2)	241 (79.5)	0.466
RAS blocker	45 (12.4)	56 (18.5)	0.028
Statin	156 (42.9)	153 (50.5)	0.049

Numbers are represented as mean ± SD or *n* (%). *VA* Vasospastic angina, *BMI* Body mass index, *GFR* Glomerular filtration rate, *LDL* Low-density lipoprotein, *HDL* High-density lipoprotein, *DHP* Non-dihydropyridine, *RAS* Renin-angiotensin system

## Discussion

Using the nation-wide database, our study sought to find out sex differences in the clinical characteristics and long-term clinical outcomes of VA. Our main findings are as follows: (1) most of the patients diagnosed with VA were male; (2) male VA patients were younger, were more frequently smoker and alcohol drinker, and had more organic stenosis; (3) there was no significant sex difference in long-term cardiovascular outcomes; and (4) concomitant significant organic coronary stenosis was independent risk factor for worse prognosis in both sexes, and higher BMI was associated with better outcomes in females among VA patients.

### Previous studies on sex differences in VA

Only a few studies have addressed sex differences in clinical characteristics and outcomes of VA [13–17]. In those studies, VA prevalence was significantly higher in males than in females: the range of proportion of females ranged from 12.7 to 24.0% [13–17]. The proportion of females was 37.9% in our study, which was higher in our study compared to previous studies. This might be due to the fact that we enrolled even patients with intermediate VA. Considering only patients with definite VA, the proportion of females was 26.5%, similar to previous studies. Sueda et al. [16] investigated 204 Japanese patients with VA, and first reported less concomitant organic lesion and fewer focal spasm in female patients compared to male patients. In another study of 104 VA patients in Korea, there were no significant difference in clinical characteristics except for lower rate of smoking and alcohol consumption in female patients [17]. Zhu et al. [14] analyzed 209 Chinese patients with VA and demonstrated that female patients had a higher incidence of ventricular fibrillation during VA attack. Those small cross-sectional studies may be insufficient to show sex differences in VA clinical features. Two recent studies of a large number of VA patients analyzed the prognosis and clinical characteristics of VA [13, 15]. A Japanese study of 1429 VA patients showed that female patients were characterized by old age, lower incidence of smoking, and less significant organic stenosis compared to male patients, and that the clinical outcomes of VA were similar between sexes [15]. Similarly, a Korean study of 986 VA patients demonstrated that female patients were younger and had a lower incidence of smoking or organic coronary stenosis than male patients [13]. In the same study, during the median follow-up period of 4.4 years, there were no significant differences in the occurrence of cardiovascular events between sexes [13]. Collectively, in most previous studies [9, 13–15, 17] and ours, VA prevalence is higher in males, and the more frequent cigarette smoking and alcohol drinking, and the greater the presence of fixed coronary lesions are common clinical feature of male VA patients compared to female patients. Also, cardiovascular outcomes of VA patients are similar between sexes [13, 15], which is in line with our results.

Sex differences in ECG changes are interesting. ST elevation was more frequently observed in men, which is the similar finding of a Japanese study [15]. Given that the rate of definite spasm was higher in men (Table 1), more frequent ST elevation may suggest more severe spasm in men. Underlying pathophysiology of more T inversion in women is still unknown. Further researches are needed to elucidate the mechanisms.

### Underlying pathophysiology

Cigarette smoking and alcohol drinking are established risk factors for coronary spasm [2, 4, 22, 24], which may be an important factor explaining the higher prevalence of VA in men. Also, male VA patients more frequently have organic coronary stenosis [9, 13–15, 17], which could affect the development of coronary artery spasm, because it is more likely to occur at the site of the coronary artery with atherosclerosis [25, 26]. Previous studies [13, 15] and ours consistently showed that the occurrence of cardiovascular events during the long-term clinical follow-up was not different between sexes. In our study, more frequent organic coronary lesions and more serious cardiovascular events associated with VA at initial attack were observed in males; however, long-term prognosis was not worse compared to females. This lack of sex difference may have been attributed to the fact that female patients were older than male patients in our study. In addition, male patients were prescribed more antiplatelets, RAS blockers, and statins at discharge from the hospital, which may have contributed to improving patient’ prognosis. Moreover, there could be the possibility that the use of vasodilators was effective and that the incidence of clinical events was too low to reach statistical significance. It is also necessary to consider that non-traditional risk factors (e.g., autoimmune and inflammatory disease, etc.), women-specific risk factors (e.g., polycystic ovary syndrome, reproductive hormones, and pregnancy associated factors such as preterm delivery, gestational diabetes, gestational hypertension, preeclampsia and eclampsia, etc.), or emotional stress have influenced the prognosis of women [27, 28]. Longer period follow-up studies of larger numbers of patients may be needed to clarify sex differences in VA clinical outcomes.

### Sex-specific prognostic factors

Sex-specific prognostic factors for VA have not been clearly identified. In Japan, smoking, history of myocardial infarction, cardiac arrest, and organic coronary stenosis in male patients, as well as age and ventricular arrhythmia in female patients were associated with the occurrence of cardiovascular events during the long-term clinical follow-up [15]. In Korea, the high-sensitivity C-reactive protein level was an independent predictor of long-term clinical outcomes in male patients with VA, but not in female patients with VA [13]. The present study showed that significant organic coronary stenosis was an independent prognostic factor of VA in both sexes, which is in line with results of previous studies [4, 15, 29, 30]. Interestingly, the novel finding of our study is that lower BMI was associated with worse clinical outcome in female with VA. Obesity is well known as a risk factor of atherosclerotic coronary artery disease in both males and females [31, 32], but its role in VA is still unknown. In our study, there is the possibility that less alcohol drinking, and more use of cardiovascular protective medications such as antiplatelets, renin-angiotensin system blockers, and statin, could at least in part play a role in improving VA prognosis in obese female patients (Table 4). In addition, increased estrogen production through aromatization of androgens in adipose tissue might play a role in more cardiovascular protection in obese women [33]. Further investigations into the sex-specific impact of BMI on the long-term clinical outcomes in VA patients are warranted.

### Study limitations

This study has several limitations. First, due to the relatively small number of study patients, we analyzed not only the patients with definite diagnosis of VA but also those with intermediate diagnosis of VA. However, when we further analyzed only patients with definite diagnosis of VA, the main results were the same, showing that there was no sex difference in clinical outcome. Nevertheless, considering the low incidence of clinical events, it may be possible that sex differences in clinical outcome did not reach statistical significance due to the small number of patients or the short duration of clinical follow-up. Second, for the similar reason, some of the important variables, such as cardioprotective medications, were not controlled for during the multivariable analysis. Third, information on menopausal status in females was unavailable in our study, which would provide additional insight into sex differences in VA. Fourth, there was lack of information on drug adherence during clinical follow-up, which may have an impact on prognosis. Lastly, as our study patients were all Koreans; generalization of our result to other ethnic groups may be difficult.

## Perspectives and significance

Although interest in sex differences in cardiovascular disease continues to increase, sex differences for VA are not well-known. Understanding sex differences is very important to improve patient prognosis through appropriate treatment. We showed that clinical outcome of VA is similar in spite of some different clinical characteristics between sexes. Significant organic coronary stenosis in both sexes and low BMI (< 25 kg/m^2^) in females were associated with worse prognosis in VA patients. Aggressive management of combined organic stenosis should be emphasized in VA patients. In female VA patients with low BMI (< 25 kg/m^2^), more attention should be paid to active treatment and monitoring to improve their clinical outcome.

## Conclusions

Although there were several sex differences in clinical characteristics such as younger age, more frequent cigarette smoking and alcohol drinking, and more occurrence of concomitant organic coronary stenosis in males than in females, there was no sex different in long-term clinical outcome in VA patients. Concomitant organic stenosis was an independent predictor for long-term clinical outcome in both sexes. Aggressive management of concomitant organic stenosis should be emphasized in VA patients. A greater BMI was associated with better outcome only in female VA patients. Further studies on the sex-specific effects of BMI on prognosis in VA patients are needed.

## Data Availability

The data that support the findings of this study are available from the corresponding author upon reasonable request.
